# 3-hydroxyanthranic acid increases the sensitivity of hepatocellular carcinoma to sorafenib by decreasing tumor cell stemness

**DOI:** 10.1038/s41420-021-00561-6

**Published:** 2021-07-06

**Authors:** Guifang Gan, Zhaopeng Shi, Dan Liu, Shaoyi Zhang, Hui Zhu, Yugang Wang, Jun Mi

**Affiliations:** 1 Shanghai Ninth People’s Hospital, Department of Clinical Laboratories, Shanghai Jiao Tong University School of Medicine, 200011 Shanghai, China; 2 Basic Medical Institute, Hongqiao International Institute of Medicine, Tongren Hospital, Key Laboratory of Cell Differentiation and Apoptosis of Chinese Ministry of Education, Shanghai Jiao Tong University School of Medicine, 200025 Shanghai, China; 3 Department of Radiation Oncology, Shandong Cancer Hospital affiliated to Shandong University, Shandong Academy of Medical Sciences, 250117 Jinan, Shandong China; 4 Department of Gastroenterology, Tongren Hospital, Shanghai Jiao Tong University School of Medicine, 200025 Shanghai, China; 5 Department of Nuclear Medicine, Rui Jin Hospital, Shanghai Jiao Tong University School of Medicine, 200025 Shanghai, China

**Keywords:** Cancer therapeutic resistance, Cancer stem cells

## Abstract

Sorafenib is the FDA-approved first-line target drug for HCC patients. However, sorafenib only confers 3–5 months of survival benefit with <30% of HCC patients. Thus, it is necessary to develop a sensitizer for hepatocellular carcinoma (HCC) to sorafenib. Here, we report that in representative HCC cell lines (SMMC-7721 and PLC8024) that are insensitive to sorafenib, 3-HAA (50 μM) significantly enhances cell sensitivity to sorafenib to an extent that could not be explained by additive effects. In nude mice carrying HCC xenograft, tumor growth is inhibited by sorafenib (10 mg/kg/day) or 3-HAA (100 mg/kg/day) alone. When used in combination, the treatment effectively prevents the xenograft from growing. In a set of mechanistic experiments, we find enhanced AKT activation and increased proportion of CD44^+^CD133^+^ cells in sorafenib-resistant HCC cells and tissues. The proportion of CD44^+^CD133^+^ cells is reduced upon 3-HAA treatment in both cultured cells and mouse xenografts, suggesting that 3-HAA could decrease the stemness of HCC. We also detect decreased phosphorylation of AKT, a regulator of the GSK3β/β-catenin signaling upon 3-HAA treatment. The AKT activator SC79 activates GSK3 β/β-catenin signaling while the Wnt inhibitor XAV-939 abolishes 3-HAA inhibition of HCC growth in vitro and in mice. The current study demonstrates that 3-HAA sensitizes HCC cells to sorafenib by reducing tumor stemness, suggesting it is a promising molecule for HCC therapy.

## Introduction

Hepatocellular carcinoma (HCC) is the fifth most prevalent primary cancer and the third leading cause of cancer-related death worldwide [1]. Most HCC patients are at the advanced stage upon diagnosis and ineligible for surgical resection, liver transplantation, or curative ablations. Even in patients receiving curative resection, ~70% of patients develop tumor recurrence within 5 years [2, 3]. Sorafenib is the FDA-approved tyrosine kinase inhibitor (TKI) for HCC patients, especially those patients with recurrent or advanced HCC since 2008 [4]. After more than a decade, novel therapeutic options for patients with advanced HCC were notably increased in the last 10 years. A couple of new drugs including Regorafenib, a derivative of Sorafenib, and Nivolumab, an immune checkpoint inhibitor, have been approved by the FDA as second-line treatments for Sorafenib-resistant HCC [5, 6]. Most recently, another TKI Lenvatinib has shown a comparable survival benefit with Sorafenib in a randomized phase III clinical trial and has also been approved by FDA as a new first-line treatment for HCC in August 2018 [7]. Metronomic capecitabine (MC) has been tested as in first and in second-line treatment for HCC patients by several studies, which demonstrated a good anticancer activity as well a very low rate of toxicities. Also, MC may be effective and well-tolerated in recurrent HCC after liver transplantation and seems a safe option for Child–Pugh B-HCC patients [8]. The combination atezolizumab plus bevacizumab has shown a survival benefit over sorafenib monotherapy, thus starting the era of immune-based combinations in HCC patients [9, 10]. Despite these encouraging advancements, the treatment options for advanced HCC patients remain very limited and further development of new therapeutic regimens is warranted [9].

Sorafenib targets multiple tyrosine kinases, including RAF, VEGFR, and PDGFR, to suppress their downstream proliferation and survival signaling pathways. However, sorafenib only confers 3–5 months of survival benefit with <30% of HCC patients sensitive to sorafenib therapy [4]. The mechanisms underlying HCC resistance to sorafenib are still not elucidated [11–13]. High-throughput forward genetic screening approaches have been widely applied to study the molecular mechanisms associated with specific cellular phenotypes, including drug resistance in human cancers. PHGDH is identified as an attractive target to overcome TKI drug resistance in hepatic cell carcinoma (HCC) by a genome-wide CRISPR/Cas9 knockout library screening and chemical genetics [14]. In our previous study, the integrated analysis of principal component analysis, gene ontology (GO), and KEGG analysis following RNA-sequencing revealed that PI3K/Akt, focal adhesion, neuroactive ligand/receptor, cytokine/cytokine receptor, and cAMP-signaling pathways were mostly regulated in sorafenib-resistant cells [15]. Cancer stem cells (CSCs) or tumor-initiating cells (TICs) have been proposed as a critical mediator of resistance to antitumor therapies in advanced HCC [16]. Current chemotherapies for advanced HCC could effectively reduce the bulk of the tumor but are unable to precisely target the HCC stem cells, resulting in a transient tumor volume reduction with a quick recurrence [17, 18]. Numerous studies showed that HCC stem cells which are epithelial cell adhesion molecules (EpCAM) positive, or label-retaining are more resistant to sorafenib [18]. Other stem cell markers of HCC easily resistant to sorafenib include CD44^+^ or CD44^+^CD133^+^ subpopulations, suggesting liver CSCs play a critical role in sorafenib resistance [11, 19]. Thus, targeting CSCs is a promising approach as it could circumvent the development of sorafenib resistance and synergizes with sorafenib to suppress HCC.

It is well known that Wnt/β-catenin signaling is critical for stem cells self-renewal [20, 21], and suppressing Wnt/β-catenin signaling can eliminate sorafenib-resistant stem-like cells [22, 23]. The phosphoinositide 3-kinase (PI3K)/AKT pathway regulates a large number of molecules involved in cancer progression and stemness, including HCC [24]. Moreover, our current study showed that sorafenib-activated AKT signaling and the levels of phosphorylated AKT increased in sorafenib-resistant HCC cells and tissues, which was consistent with previous findings [24–26]. Inhibition of AKT activity reversed the acquired resistance of HCC to sorafenib [27, 28]. 3-hydroxyanthranilic acid (3-HAA), a derivative of kynurenine, suppressed AKT activity in HCC. Thus, we employed 3-HAA to investigate whether 3-HAA could sensitize HCC cells and xenografts to sorafenib by decreasing tumor stemness.

## Results

### Tumor stemness is increased in sorafenib-resistant HCC cells

**Fig. 1:**
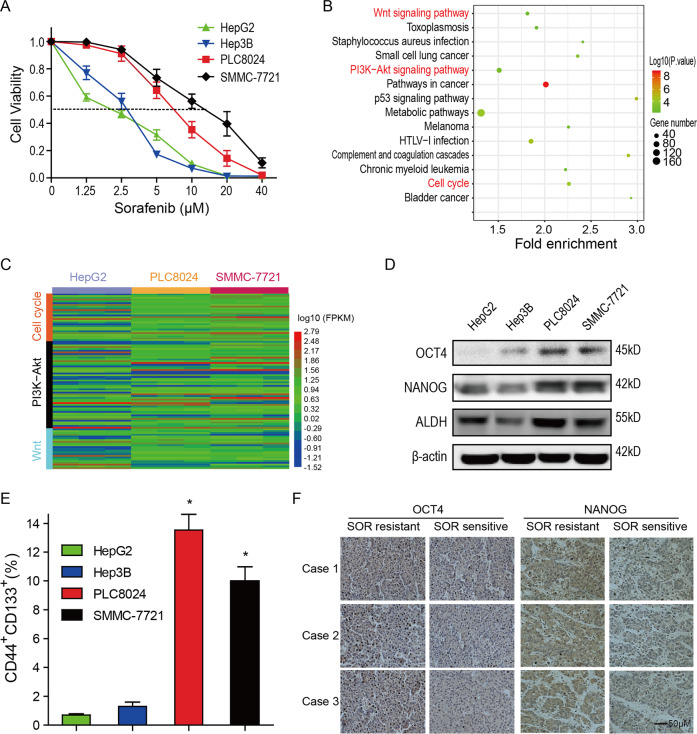
Tumor stemness is increased in sorafenib-resistant hepatocellular carcinoma cells. **A** SMMC-7721 and PLC8024 cells were more resistant to sorafenib than HepG2 and Hep3B cells. HCC cells were treated with 0–40 μM sorafenib and the cell viability was analyzed by the CCK8 assay. Data were presented as mean ± SD (*n* = 3). **B** The KEGG analysis on the upregulated/downregulated genes. The genes expression changed more than 2 folds were analyzed between sorafenib-resistant vs. sorafenib-sensitive HCC cells. **C** Heatmap of gene expression participated in cell cycle pathway, Wnt signaling, and PI3K-AKT pathway. The levels of the gene expression were reflected by different colors, colors from blue to red stood for expression levels from low expression to high expression. **D** The expression of stem cell markers was enhanced in sorafenib-resistant HCC cells. The expression of ALDH, SOX2, and OCT4 was analyzed. **E** The CD44^+^CD133^+^ subpopulations were increased in sorafenib-resistant HCC cells. The CD44^+^CD133^+^ subpopulation was analyzed by flow cytometry using anti-CD44 and anti-CD133 antibodies. *P < 0.05, **P < 0.01. **F** Stemness was increased in sorafenib-resistant HCC tumors. OCT4 and NANOG expression were detected by IHC. Bar = 100 μm.

CSCs or TICs have been proposed as key mediators of HCC resistance to anti-tumor therapies including sorafenib. To determine the mechanism by which HCC cells possess the inherited resistance to sorafenib, the sensitivity of four classical HCC cell lines (HepG2, SMMC-7721, PLC8024, and Hep3B) to sorafenib was first evaluated by the cell viability assay. As shown in Fig. 1A, sorafenib inhibited the growth of all four HCC cell lines, but PLC8024 and SMMC-7721 cells were apparently more resistant to sorafenib treatment than HepG2 and Hep3B cells. The IC_50_ values of sorafenib were 8.77 and 18.08 µM to PLC8024 and SMMC-7721 cells, respectively, whereas those were 4.92 and 3.26 µM to Hep3B and HepG2 cells. As HepG2 was most sensitive to sorafenib treatment determined by CCK8 assay, HepG2 was chosen as a representative sorafenib-sensitive HCC cell line. The KEGG analysis on the common upregulated/downregulated genes between the two groups of HCC cells displayed that the Wnt pathway, the PI3K/Akt pathways, and the cell cycle pathway were activated/inactivated in sorafenib-resistant cells (Fig. 1B). The expression of genes involved in three pathways showed that the Wnt signal and the PI3K/AKT signal were activated in the sorafenib-resistant HCC cells (Fig. 1C), both pathways are critical for the self-renewal of progenitor/stem cells.

Moreover, the expression of stem cell markers including OCT4, NANOG, and ALDH was analyzed to determine whether stemness was enhanced in sorafenib-resistant HCC cells. As shown in Fig. 1D, the expression of critical stemness markers was markedly increased in PLC8024 and SMMC-7721 cells than in HepG2 and Hep3B cells. The flow cytometry results confirmed that the stemness in PLC8024 and SMMC-7721 cells was higher than that in HepG2 and Hep3B cells, evidenced by the increased CD44^+^/CD133^+^ subpopulations, which were 13.5 ± 1.66% and 9.6 ± 0.78% in PLC8024 and SMMC-7721 cells, respectively, while these were 0.71 ± 0.23% and 1.25 ± 0.13% in HepG2 and Hep3B cells, respectively (Figs. 1E and S1A). Furthermore, the stemness markers OCT4 and NANOG were found to be overexpressed in sorafenib-resistant tumors (Fig. 1F). These observations suggested that stemness is enhanced in sorafenib-resistant HCC cells.

### Wnt signaling is activated in sorafenib-resistant HCC cells

**Fig. 2:**
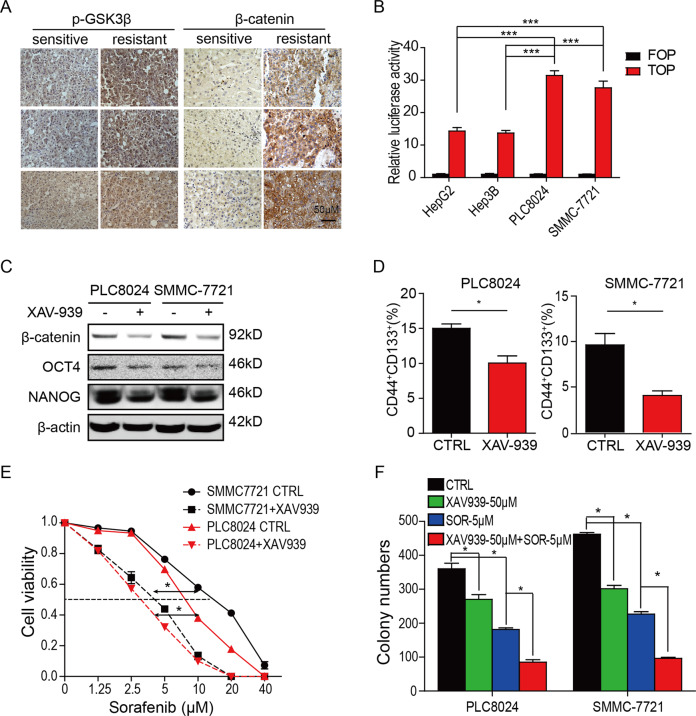
Wnt signaling is activated in sorafenib-resistant hepatocellular carcinoma cells. **A** Wnt signaling pathway was activated in sorafenib-resistant HCC tumors. Bar = 100 μm. **B** β-catenin activity was increased in sorafenib-resistant HCC cells. The TOP/FOP-Flash reporter assay was performed. **C** The Wnt inhibitor XAV-939 alleviated the expression of OCT4 and NANOG in sorafenib-resistant HCC tumors. Cells were treated by XAV-939 at the indicated dose for 2 days. **D** XAV-939 decreased the proportion of CD44^+^CD133^+^ subpopulation in sorafenib-resistant HCC tumors. The cells were treated by 50 μM XAV-939 for 2 days. *P < 0.05. **E** XAV-939 inhibits cell growth of sorafenib-resistant HCC cells. The cell viability of HCC cells was analyzed by CCK8. Data were presented as mean ± SD (*n* = 3). *P < 0.05. **F** XAV-939 reduced colony formation in sorafenib-resistant SMMC-7721 and PLC8024 cells. The cells were treated with 50 μM of XAV-939 alone or 5 μM of sorafenib alone or the combination. *P < 0.05.

Wnt/β-catenin signaling is critical for the self-renewal of progenitor/stem cells. Above data showed the Wnt pathway was activated in sorafenib-resistant HCC (Fig. 1B), thus, the protein level of β-catenin and the phosphorylation level of GSK-3β were analyzed to identify whether the Wnt signaling activated in sorafenib-resistant HCC cells. As shown in S2A, the expression of β-catenin and the phosphorylation of GSK3β increased in sorafenib-resistant HCC cells. Also, the IHC staining showed that the protein level of β-catenin and the phosphorylation of GSK3β were elevated in sorafenib-resistant HCCs compared to sorafenib-sensitive tissues (Fig. 2A). Moreover, the β-catenin activity also increased in sorafenib-resistant HCC compared to the sensitive cells, measured by the TOP/FOP-Flash reporter assay (Fig. 2B), suggesting that the Wnt signaling activated in sorafenib-resistant HCCs.

To determine whether Wnt/β-catenin signaling regulates the sensitivity of HCC cells to sorafenib, the Wnt inhibitor XAV-939 was employed to test whether the Wnt signaling is critical for the sorafenib resistance of HCC cells. As shown in Fig. 2C, the XAV-939 decreased the protein level of β-catenin and reduced the stemness marker levels of OCT4 and NANOG. Consequently, the XAV-939 decreased the CD44^+^CD133^+^ subpopulation and increased the sensitivity of SMMC-7721 and PLC8024 cells to sorafenib (Fig. 2D, E, S2B). CCK8 assay showed that The IC_50_ values of sorafenib reduced from 8.366 to 3.20 µM and 17.78 to 4.90 µM in PLC8024 and SMMC-7721 cells, respectively (Fig. 2E). Moreover, colony formation assay verified that the XAV-939 promoted the inhibition of sorafenib on SMMC-7721 and PLC8024 cells (Figs. 2F and S2C). These observations suggested that the Wnt/β-catenin signaling increased sorafenib resistance by enhancing the stemness of HCCs.

### 3-hydroxyanthranic acid inhibits tumor stemness of sorafenib-resistant HCC cells

**Fig. 3:**
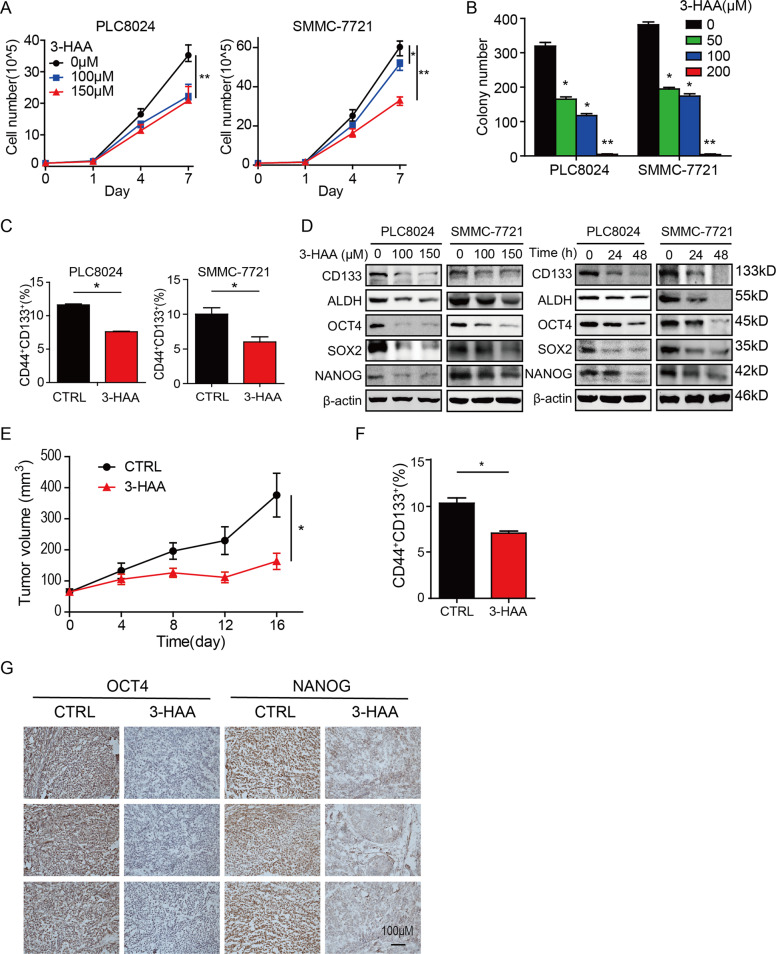
3-hydroxyanthranic acid inhibits tumor stemness of sorafenib-resistant HCC cells. **A** 3-HAA inhibited cell growth of sorafenib-resistant HCC cells. Cell numbers of HCC cells were counted by cytometry. Data were presented as mean ± SD (*n* = 3). *P < 0.05, **P < 0.01. **B** 3-HAA reduced colony formation in sorafenib-resistant SMMC-7721 and PLC8024 cells. Cells were treated with 3-HAA at the indicated dose. *P < 0.05, **P < 0.01. **C** The ratio of CD44^+^CD133^+^ subpopulation was decreased in 3-HAA-treated SMMC-7721 and PLC8024 cells. Cells were treated with 3-HAA at the dose of 100 μM for 48 h. *P < 0.05. **D** 3-HAA inhibited the expression of stem cell markers in HCC cells. The treating time was 48 h for the dose–response experiment and the dose of 3-HAA was 100 μM for the time-course analysis. **E** 3-HAA suppressed tumor growth of SMMC-7721 xenografts. The 3-HAA was administrated 100 mg/kg day by intraperitoneal injection for 16 days, five mice were recruited in the control and 3-HAA-treated group. The tumor volumes were presented as mean ± SD. *P < 0.05. **F** 3-HAA decreased the proportion of CD44^+^CD133^+^ subpopulation in SMMC-7721 xenografts. Tumor cells were analyzed by flow cytometry using anti-CD133-PE and anti-CD44-APC. *P < 0.05. **G** 3-HAA inhibited the expression of stem cell markers in SMMC-7721 xenografts. Bar = 100 μm.

Our previous research demonstrated 3-HAA, a derivative of kynurenine, inhibited HCC growth. To determine whether 3-HAA inhibits HCC growth through regulating the stemness of HCC, the effects of 3-HAA on tumor stemness were performed in vitro and in vivo. The cell viability and colony formation ability were first analyzed. As shown in Fig. 3A, 3-HAA did inhibit the growth of sorafenib-resistant PLC8024 and SMMC-7721 cells in a dose-dependent manner. Also, 3-HAA significantly decreased the colony numbers of PLC8024 and SMMC-7721 cells (Figs. 3B and S3A). Moreover, 3-HAA decreased the ratio of CD133^+^/CD44^+^ subpopulations in total PLC8024 and SMMC-7721 cells from 12.6% to 7.6% and from 9.7% to 6.2%, respectively (Figs. 3C and S3B).

To further determine whether 3-HAA inhibits the stemness of HCC cells, the expression of stem cell markers including CD133, ALDH, SOX2, OCT4, and NANOG were analyzed. As shown in Fig. 3D, all these stemness markers were downregulated in a dose-dependent and time-dependent manner after 3-HAA treatment. Consistent with the in vitro results, 3-HAA marked inhibited tumor growth by 69.35% (Figs. 3E and S3C). Moreover, 3-HAA significantly reduced the ratio of CD133^+^CD44^+^ cells and decreased the expression of OCT4 and NANOG in xenografts (Figs. 3F, G, and S3D). These observations suggested that 3-HAA inhibits the growth and stemness of sorafenib-resistant HCC cells.

### 3-HAA suppresses Wnt signaling by decreasing AKT activity

**Fig. 4:**
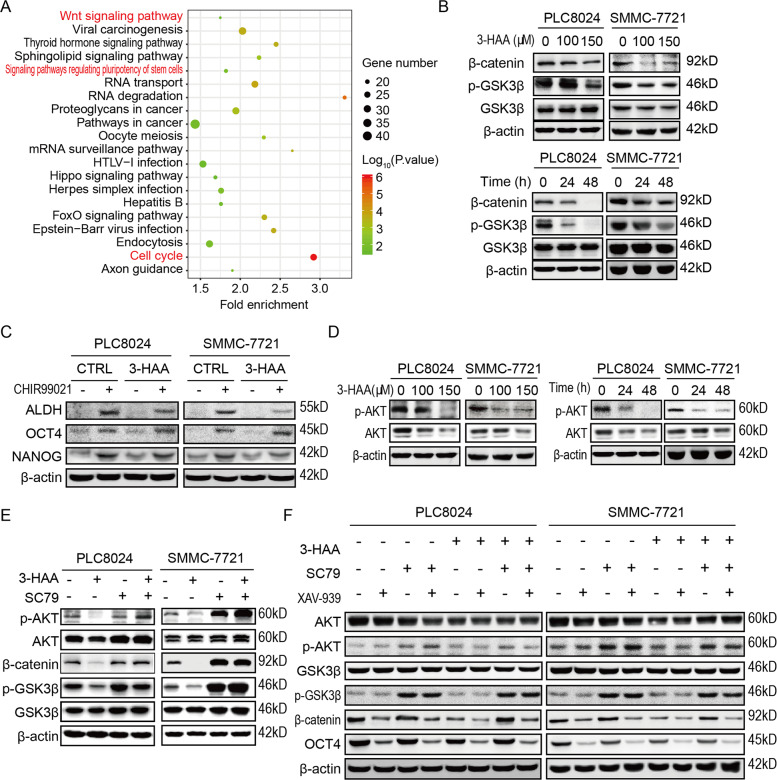
3-HAA suppressed Wnt signaling by decreasing AKT activity. **A** The KEGG analysis on the upregulated/downregulated genes. The genes whose expression changed more than 2 folds in 3-HAA-treated HCC cells were analyzed. **B** 3-HAA inhibited GSK3β/β-catenin signaling in SMMC-7721 and PLC8024 cells. The treating time for the dose course experiment was 24 h. The 3-HAA dose for the time course analysis was 100 μM. **C** The Wnt activator CHIR99021 abolished 3-HAA inhibition on the expression of stem cell markers ALDH, OCT4, and NANOG. The dose of CHIR99021 and 3-HAA was 5 and 100 μM, respectively. Cells were treated for 24 h. **D** AKT phosphorylation detection in 3-HAA-treated SMMC7721 and PLC8024 cells. The treating time and the 3-HAA dose were the same as above. **E** SC79 recovered 3-HAA’s inhibition on AKT phosphorylation and GSK3β/β-catenin signaling. The dose of SC79 and 3-HAA were 15 and 100 μM, respectively. Cells were treated for 24 h. **F** CHIR99021 abolished 3-HAA inhibition on AKT phosphorylation and GSK3β/β-catenin signaling. The dose of CHIR99021 and 3-HAA was 5 and 100 μM, respectively. Cells were treated for 24 h.

To explore the mechanism by which 3-HAA inhibiting stemness in HCC cells, the KEGG analysis was performed on the differential expressed genes of SMMC-7721 and PLC8024 cells treated with or without 3-HAA. As shown in Fig. 4A, the Wnt signaling, the pathway regulating pluripotency of stem cells, and the cell cycle pathway were markedly changed, suggesting 3-HAA may inhibit stemness of HCC cells by regulating Wnt signaling. Indeed, 3-HAA inhibited Wnt/β-catenin signaling in a dose- and time-dependent manner (Fig. 4B), while showed little influence on Hedgehog or Notch signaling (Fig. S4A, B). Meanwhile, the CHIR99021, an activator of Wnt, rescued the 3-HAA-mediated suppression of cell growth (Fig. S4C) and recovered the expression of stem cell markers suppressed by 3-HAA treatment in HCC cells (Fig. 4C).

The Wnt/β-catenin signaling could be regulated by the AKT, which was activated in sorafenib-resistant HCC cells (Fig, 1B). Therefore, the effect of 3-HAA on AKT phosphorylation was determined in sorafenib-resistant HCC cells. Immunoblotting assays revealed that 3-HAA decreased AKT phosphorylation in a time-dependent and dose-dependent manner (Fig. 4D). Meanwhile, 3-HAA had little effect on the mRNA levels of CTNNB1, a gene encoding β-catenin (Fig. S4D, E).

To determine whether AKT signaling is critical for 3-HAA regulation on stemness of HCC cells, the AKT activator (SC79) was applied to investigate the effect of AKT activation on GSK-3β/β-catenin signaling. Immunoblotting assays revealed that SC79 abolished the inhibition of Wnt/β-catenin signaling by 3-HAA (Fig. 4E). Moreover, SC79 increased 3-HAA-inhibited OCT4 expression, and the Wnt inhibitor XAV-939 restored the promoting effect of SC79 on OCT4 expression (Fig. 4F). These observations suggested that 3-HAA inhibited HCC cell stemness by suppressing AKT/GSK-3β/β-catenin signaling.

### The 3-HAA increases the sensitivity of HCC cells to sorafenib by suppressing stemness

**Fig. 5:**
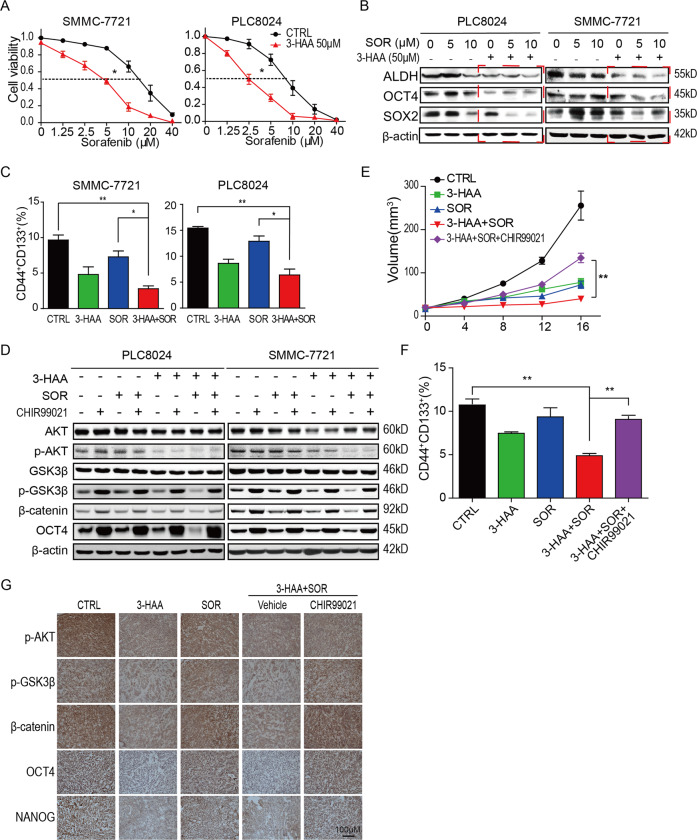
The 3-HAA increases the sensitivity of HCC cells to sorafenib by suppressing stemness. **A** The cell viability of 3-HAA-treated SMMC-7721 and PLC8024 cells was analyzed by CCK8 assay. Cells were treated with sorafenib at the indicated dose with or without 100 μM 3-HAA for 4 days. *P < 0.05. **B** The combination of 3-HAA and sorafenib dramatically inhibited ALDH, SOX2, and OCT4 expression in SMMC-7721 and PLC8024 cells. Cells were treated with 100 μM of 3-HAA and sorafenib at the indicated dose for 24 h. **C** The combination of sorafenib and 3-HAA significantly reduced the proportion of CD133^+^CD44^+^ tumor cells than either 3-HAA alone or sorafenib alone in SMMC-7721 and PLC8024 cells. Cells were treated with 100 μM 3-HAA and 5 μM sorafenib for 24 h. *P < 0.05, **P < 0.01. **D** CHIR99021 recovered the AKT/GSK3β/β-catenin signaling and the expression of stemness markers, inhibited by the combination treatment. Cells were treated with 100 μM 3-HAA, 5 μM sorafenib, and/or 5 μM CHIR99021 for 24 h. **E** CHIR99021 recovered the combination-suppressed tumor growth of SMMC-7721 xenografts. 3-HAA and sorafenib were separately administrated by intraperitoneal injection for 16 days at the dose of 100 and 10 mg/kg day, respectively. CHIR99021 was administered orally at 40 mg/kg day, five mice were recruited in each group. The tumor volumes are presented as mean ± SD. **P < 0.01. **F** CHIR99021 increased the ratio of CD44^+^CD133^+^ subpopulation in SMMC-7721 xenografts. Tumor cells were analyzed by flow cytometry using anti-CD133-PE and anti-CD44-APC. **P < 0.01. **G** CHIR99021 increased the combination-inhibited AKT/Wnt/β-catenin signal. The expression of OCT4, NANOG, p-AKT, p-GSK3β, and β-catenin were analyzed in xenografts. Bar = 100 μm.

To determine whether 3-HAA sensitizes the HCC PLC8024 and SMMC-7721 cells to sorafenib, the effects of the combination of 3-HAA and sorafenib were evaluated in sorafenib-resistant HCC cells by CCK8 assays. As shown in Fig. 5A, 50 μM of 3-HAA combined with 5 μM of sorafenib suppressed SMMC-7721 and PLC8024 cell growth to a significantly greater extent than sorafenib alone. And, 50 μM of 3-HAA showed an add-on effect in SMMC-7721 and PLC8024 cells. The IC_50_ values of sorafenib reduced from 8.77 to 3.42 µM and 18.08 to 3.95 µM in PLC8024 and SMMC-7721 cells, respectively. Moreover, the combined treatment of sorafenib and 3-HAA markedly down-regulated the expression of ALDH, SOX2, and OCT4 in PLC8024 and SMMC-7721 cells while sorafenib alone did not significantly reduce protein levels of these HCC stemness markers (Fig. 5B).

To determine whether CSCs were enriched in sorafenib-treated HCC cells, the ratio of CD44^+^CD133^+^ subpopulations was analyzed by flow cytometry. The combination of sorafenib and 3-HAA significantly reduced the proportion of CD133^+^CD44^+^ tumor cells than either sorafenib or 3-HAA alone (Figs. 5C and S5A). However, the Wnt activator CHIR99021 restored 3-HAA-decreased phosphorylation of AKT and GSK3β, and consequently increased the protein level of β-catenin and OCT4 (Fig. 5D). In addition, animal studies revealed that the combination of sorafenib (10 mg/kg day) and 3-HAA (100 mg/kg day) caused xenograft growth delay and CHIR99021 abolished the synergistic effect of 3-HAA and sorafenib on tumor growth (Figs. 5E and S5B). Furthermore, the combined treatment reduced the proportion of CD133^+^CD44^+^ tumor cells than either sorafenib alone or 3-HAA alone. However, additional CHIR99021 recovered the proportion of CD133^+^CD44^+^ subpopulation in xenografts (Figs. 5F and S5C). The immunohistochemistry staining demonstrated that the combined treatment inhibited AKT/Wnt/β-catenin signal and reduced the tumor stemness in HCC xenografts, while the Wnt activator CHIR99021 restored the Wnt/β-catenin signal and tumor stemness (Fig. 5G), suggesting that the AKT/GSK3β/β-catenin signaling was critical for the synergistic effect of the combination treatment on HCC cells.

## Discussion

The discovery of sorafenib provides hope for combating HCC, but this promising treatment only demonstrated limited survival benefits, reflected by a low response rate to sorafenib (de novo resistance) and quick recurrence after initial response (acquired resistance) [4, 13]. Therefore, it is necessary to develop novel therapeutic agents that can overcome acquired or de novo resistance to sorafenib which could lead to the discovery of promising strategies to increase therapeutic efficacy against HCC [12]. The current study demonstrated that 3-HAA, a structural analog of kynurenine, could sensitize HCC cells to sorafenib both in vitro and in vivo*.* This tumor-growth inhibitory effect of 3-HAA was at least partially attributed to its inhibition of stemness of HCC cells via suppressing AKT/GSK-3β/β-catenin signaling (Fig. 4). Our findings provide the first proof of concept that adding a kynurenine derivative to sorafenib therapy could lead to synergistic suppression of HCC growth both in vitro and in vivo.

Cancer stem/initiating cells (CSC/CIC) are considered to initiate tumors and mediate tumor growth and drug resistance [20]. Thus, discovering effective therapeutic methods against CSC that are commonly resistant to standard therapies is an attractive approach for HCC [18]. Liver CSCs, represented by CD44, CD133, EpCAM, or CD90-positive cells, have been proposed to be a key regulator of sorafenib resistance in HCC [11, 18, 29]. Through comprehensive genomic sequencing, mutations affecting the Wnt/β-catenin pathway have been found in 26–40% of HCC cases [21, 30, 31], and linked to CSCs, suggesting their importance in maintaining the CSC phenotype [19, 32]. Our data showed that Wnt/β-catenin signaling is activated in sorafenib-resistant HCC cells and clinical samples [30, 31], and suppressing Wnt/β-catenin signaling can reduce the number of sorafenib-resistant cells [22], supporting our hypothesis that HCC stem cell is responsible for sorafenib-resistance.

Moreover, multiple pieces of evidence suggest that AKT activation in CSCs is a key mechanism by which CSCs mediate chemoresistance, a common hallmark of CSC [24]. AKT appears to be able to drive chemoresistance through several mechanisms, including upregulation of drug transporter proteins as well as pro-survival pathways, including the Bcl-2 pathway. Activation of AKT has been shown to increase ABCG2-driven SP^+^ glioma tumor-initiating CSC [19, 24]. In consistent with other reports, our previous study revealed that PI3K/AKT was mostly regulated in sorafenib-resistant cells using RNA-sequencing [15] and concomitantly enhanced GSK3β/β-catenin signaling [21, 25, 32], suggesting that AKT activation increases the stemness of HCC cells.

Although therapeutic options including multikinase inhibitors, immune checkpoint inhibitors (ICIs), and combinations of both strategies, have recently increased for patients with advanced HCC [5–7], multikinase inhibitors currently used in patients with HCC, such as sorafenib, regorafenib, and lenvatinib, have lower response rates and higher therapeutic resistance than targeted therapy agents in other cancers. Indeed, driver oncogenes have not yet been accurately identified or used for HCC therapy. In this study, we only determined the add-on effect of 3-HAA on Sorafenib treatment, did not test the effect of 3-HAA on other inhibitors. In the future study, we will determine whether 3-HAA could increase the sensitivity of HCC to other inhibitors, such as Regorafenib, Nivolumab, and atezolizumab.

In addition, 3-HAA is a structural analog of kynurenine, and both are derived from essential amino acid tryptophan. Recent studies showed that kynurenine promoted tumor progression and immune evasion through binding to the acyl hydrocarbon receptor [33–35]. However, in our studies, we demonstrated that 3-HAA decreased the tumor stem cell subpopulation and induced HCC cell death by inhibiting tumor stemness, which is distinct from the effect of kynurenine.

Taken together, our results demonstrated that 3-HAA down-regulated AKT/GSK3β/β-catenin signaling consequently inhibited the stemness of HCC cells. Besides, 3-HAA had a synergistic effect with sorafenib against HCC, suggesting that 3-HAA is a promising molecule for HCC therapy.

## Materials and methods

### Cells

Human HCC cell line HepG2 (American Type Culture Collection; Manassas, VA, USA; RRID: CVCL_0027), Hep3B (RRID: CVCL_0326), PLC8024 (RRID: CVCL_0485) (Cell Bank of the Chinese Academy of Science, Shanghai, China) and SMMC7721 (RRID: CVCL_0336) (Genechem Co., Ltd., Shanghai, China) were grown at 37 °C in DMEM (Invitrogen, Grand Island, NY, USA) containing 5% CO_2_ atmosphere and supplemented with 10% heat-inactivated fetal bovine serum (PAA, Australia) in the presence of 100 U/mL penicillin and 0.1 mg/mL streptomycin.

### CCK8 assays

HCC cells were seeded into a 96-well plate at the density of 2000 cells per well, followed by the treatment of 3-HAA (Sigma, Cat: 148776), XAV-939 (TargetMol, Cat: T1878), and/or sorafenib (Meilunbio, Cat: MB1226) at appropriate doses as indicated elsewhere. CCK8 assays were performed in triplicates as instructed by the manufacturer (Dojindo, Tabaru, Japan). Absorbance was measured at 450 nm using a microplate reader and cell viability was normalized to control and the mean of at least three independent experiments was calculated.

### Functional annotation of the differentially expressed genes (DEGs)

The Database for Annotation, Visualization, and Integrated Discovery (DAVID, https://david.ncifcrf.gov/version 6.8) was used to perform a preliminary analysis of the obtained DEGs with systematic and comprehensive biological function notes. The Functional Annotation Tool is the core of DAVID, which includes GO enrichment and Kyoto Encyclopedia of Genes and Genomes (KEGG) analyses. Through GO enrichment analysis, we can roughly compare and classify DEGs to better understand their biological characteristics. The KEGG helps us to study the functional interpretation of genes and genomes as a whole network. In our paper, the threshold *P*-value < 0.05 was considered statistically significant.

### Xenograft assays

Six-week-old male BALB/c nude mice were obtained from Shanghai SLAC Laboratory Animal Center, Shanghai, China. All mice were maintained according to the Guide for the Care and Use of Laboratory Animals published by the National Institutes of Health, USA. SMMC7721 cells (1 × 10^6^ cells) were injected subcutaneously into the armpit area of each mouse. When the tumor volume reached 20–80 mm^3^, 100 mg/kg day 3-HAA and/or 10 mg/kg day sorafenib was administered intraperitoneally daily for 16 days. CHIR99021 (TargetMol, Cat: T2310) was administered orally at 40 mg/kg day. The tumor volume was determined at the indicated time points using digital caliper measurements and calculated by the following formula:

$${\mathrm{Tumor}}\;{\mathrm{volume}}\left( {{\mathrm{mm}}^3} \right) = \raise.5ex\hbox{$\scriptstyle 1$}\kern-.1em/ \kern-.15em\lower.25ex\hbox{$\scriptstyle 2$} \times {\mathrm{longest}}\;{\mathrm{diameter}}^2 \times {\mathrm{shortest}}\;{\mathrm{diameter}}$$

At the indicated time points, the mice were sacrificed, the tumors were excised, and tumor weight was measured.

The study protocol was approved by the Institutional Animal Care and Use Committee of Shanghai Jiao Tong University School of Medicine and animal study was carried out in accordance with established national and institutional guidelines on the use of experimental animals.

### Flow cytometry

SMMC-7721 and PLC8024 cells were treated with sorafenib or 3-HAA at appropriate doses for 48 h and harvested by trypsinization and washed with phosphate-buffered saline (PBS). The cells were then stained with anti-human CD133-PE (eBioscience, Cat: 12-1338-71) and CD44-APC (eBioscience, Cat: 17-0441-82) for 30 min. At least 1 × 10^6^ cells were analyzed by a FACS Aria II (BD Falcon, Franklin Lakes, NJ, USA). Cells were gated based on their forward and side scatter properties. Furthermore, mouse xenograft tissues were minced and digested with collagenase and DNase 1 (Life Technologies, Grand Island, NY, USA) at 37 °C for 30 min, and then filtered with 40-micron cell strainers (BD Falcon). Tumor cells were stained with anti-CD133-PE and anti-CD44-APC and analyzed by flow cytometry.

### Western blotting assays

Appropriate cells were lysed in RIPA lysis buffer containing a cocktail of protease inhibitors (Roche) and PMSF. Total protein concentration was determined using the bicinchoninic acid (BCA) assay kit (Ding Guo Biotechnology, Cat: BCA02). Antibodies against the following proteins were used for immunoblotting: NANOG (CST, Cat: 4893), ALDH (BD Pharmingen, Cat: 611195), OCT4 (CST, Cat: 2750), CD133 (CST, Cat: 64326), SOX2 (CST, Cat: 2748), phospho-AKT (Thr308) (CST, Cat: 2965), AKT (CST, Cat: 4691), phospho-GSK-3β (Ser9) (CST, Cat: 9323), GSK-3β (CST, Cat: 9315), β-catenin (CST, Cat: 8480, and β-actin (Santa Cruz Biotechnology, Cat: sc-47778). The immunoblots were scanned using an Odyssey infrared imaging system (LI-COR). Immunolabeling was detected using the ECL reagent (Sigma). Protein expression was normalized against β-actin.

### Real-time quantitative PCR

Total cellular RNA was prepared using the TRIzol reagent (Invitrogen) as instructed by the manufacturer and was reverse transcribed using an RT reagent kit (TAKARA, Dalian, China). After cDNA synthesis, real-time quantitative polymerase chain reaction (PCR) was performed in triplicate in a 96-well plate with an ABI7500 real-time PCR system (Life Technologies, Grand Island, NY, USA) using SYBR Green mixture. CTNNB1 expression was normalized against ⎕β-actin. The primer sequences were as follows: *CTNNB1*, forward 5′-ATGATGGTCTGCCAAGTGGG-3′ and reverse 5′-GGCCATCTCTGCTTCTTGGT-3′ and⎕⎕ β-actin, forward 5′-GCGGGAAATCGTGCGTGACATT-3′ and reverse 5′-GATGGAGTTGAAGGTAGTTTCG-3′.

### Collection of Sorafenib-resistant and sorafenib-sensitive HCC tumors

Human HCC tumors were obtained from Eastern Hepatobiliary Surgery Institute, Second Military Medical University. Sorafenib was administered at a dosage of 400 mg orally twice daily to patients with HCC according to industry recommendations. After the treatment process, sorafenib-sensitive HCC tumors showed a significant beneficial effect on the tumor burden, performance status (PS), tumor stage, and prior therapy on survival, time to progression (TTP). While the clinical course had no significant difference and was defined as sorafenib-resistant tumors.

### Immunohistochemistry

For paraffin-embedded tissues, the sections were deparaffinized with xylene, followed by rehydration through incubation with a grading concentration of ethanol/water (from 100% ethanol successively to 70% ethanol). Slides were then incubated in methanol with 3% H_2_O_2_ at room temperature. After immersion in 10 mM sodium citrate buffer at 96 °C for 15 min, the sections were blocked with 10% goat serum for 1 h, then incubated with primary antibodies at 4 °C overnight. After incubation with primary and HRP-conjugated secondary antibodies, and sections were visualized with a DAB substrate. Images were captured by microscopy.

For the frozen sections, sections were fixed in 4% (v/v) phosphate-buffered paraformaldehyde for 20 min at room temperature. Slides were then immersed into 3% H_2_O_2_, blocked with 10% goat serum, incubated with antibodies, and visualized with a DAB substrate as described above.

### Colony formation assay

PLC8024 and SMMC-7721 cells were seeded into 100 mm plastic dishes at a density of 1000 cells per well and were allowed to grow for 10–14 days until clones were visible. PBS-washed cells were fixed with 4% paraformaldehyde and stained with 0.1% crystal violet. The clones containing more than 50 cells were counted.

### TOP/FOP-Flash reporter assay

For the TOP/FOP-Flash reporter assay, the TOP/FOP-Flash reporter and pTK-RL plasmids were co-transfected into cells. Cells were harvested for analysis with the Dual-Luciferase Reporter Assay System (Promega, Madison, WI, USA) 48 h after transfection. Luciferase activity was measured using the PerkinElmer EnSpire Multilabel Reader 2300 (PerkinElmer Inc., Waltham, MA, USA). The luciferase intensity was normalized to the Renilla luciferase value for transfection efficiency.

### Statistical analysis

Data were presented as means ± SD. All data were representative of at least three independent experiments. Differences between groups were assessed by Student’s *t* test; all presented differences were *P* < 0.05 unless otherwise stated.

## Supplementary information

Supplmental figure 1

Supplmental figure 2

Supplmental figure 3

Supplmental figure 4

Supplmental figure 5
