# Supplementary figures and images for: 3-hydroxyanthranic acid increases the sensitivity of hepatocellular carcinoma to sorafenib by decreasing tumor cell stemness
Source: Cell Death Discov. 2021 Jul 6;7:173. doi: 10.1038/s41420-021-00561-6 (PMC8260721; doi:10.1038/s41420-021-00561-6)

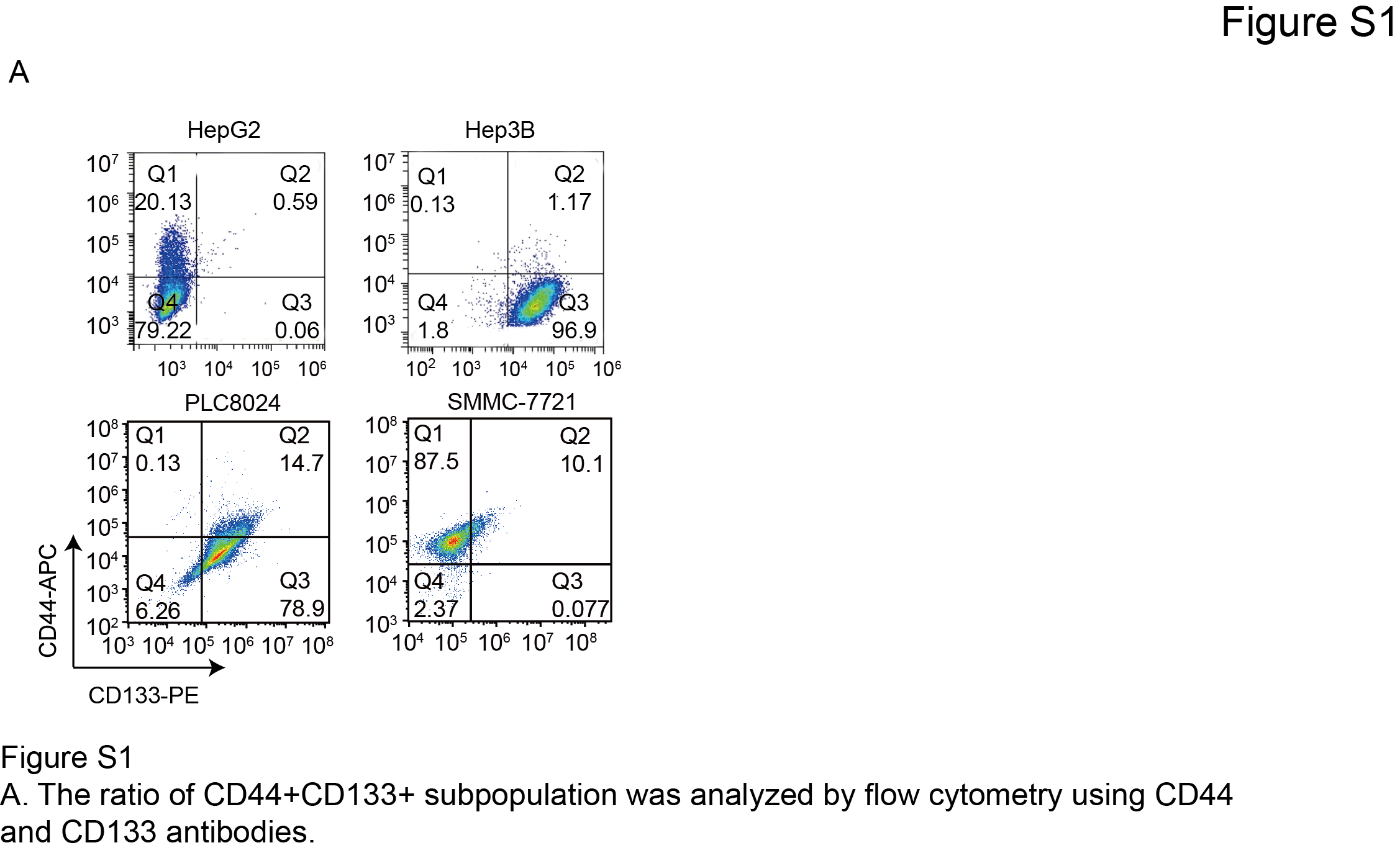

Supplement: Supplementary file 1 — Supplmental figure 1 [file 41420_2021_561_MOESM1_ESM.png]

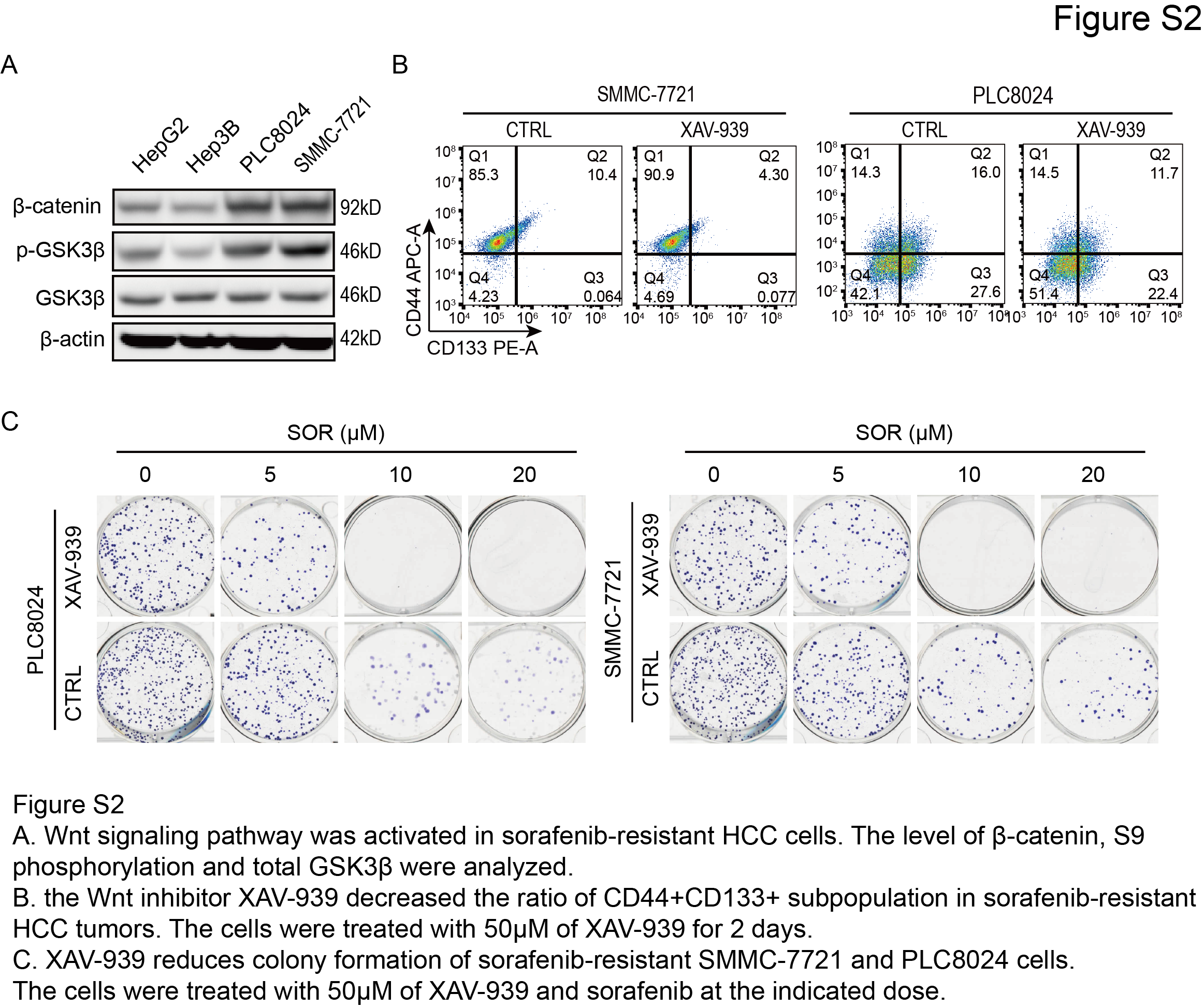

Supplement: Supplementary file 2 — Supplmental figure 2 [file 41420_2021_561_MOESM2_ESM.png]

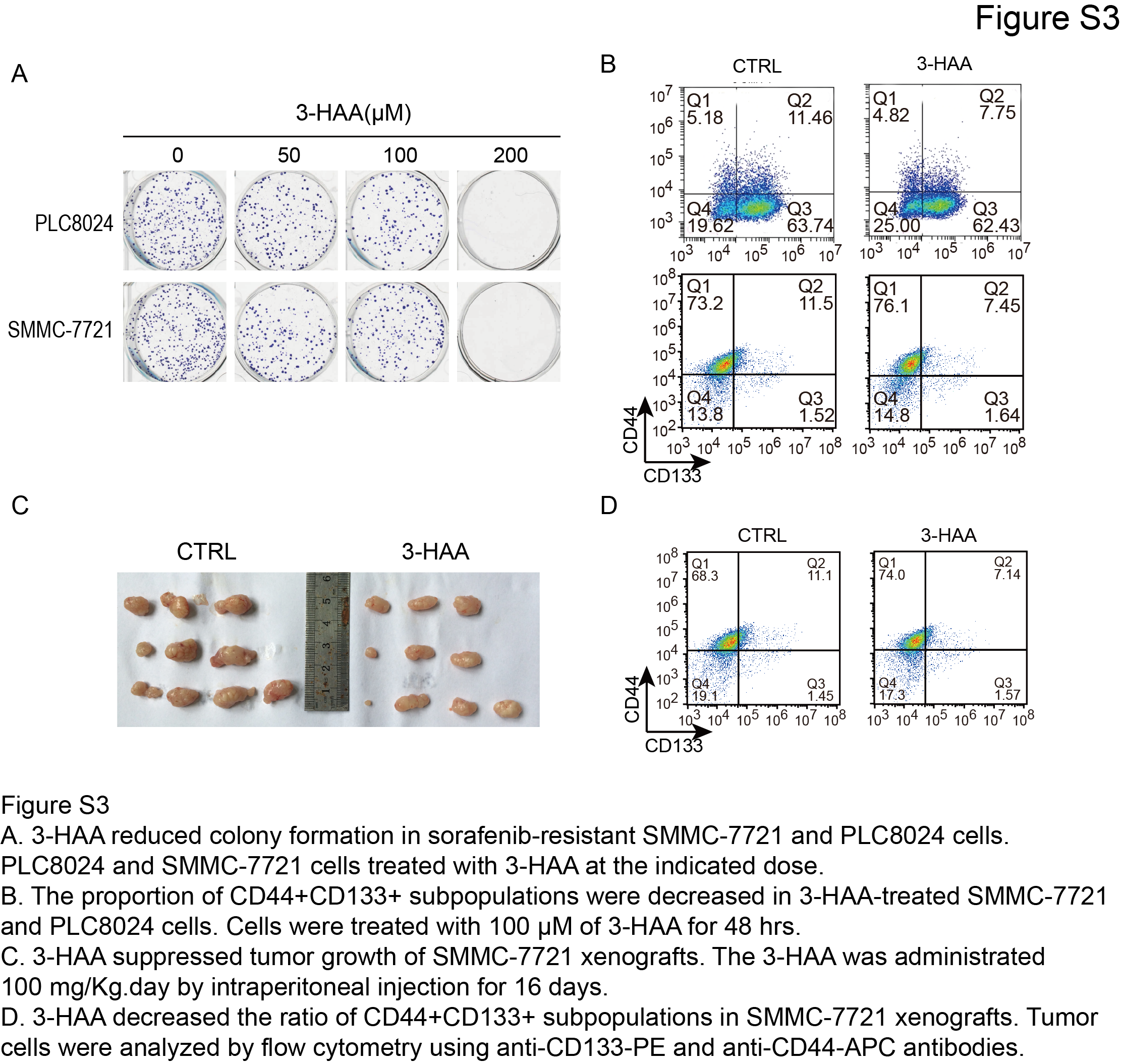

Supplement: Supplementary file 3 — Supplmental figure 3 [file 41420_2021_561_MOESM3_ESM.png]

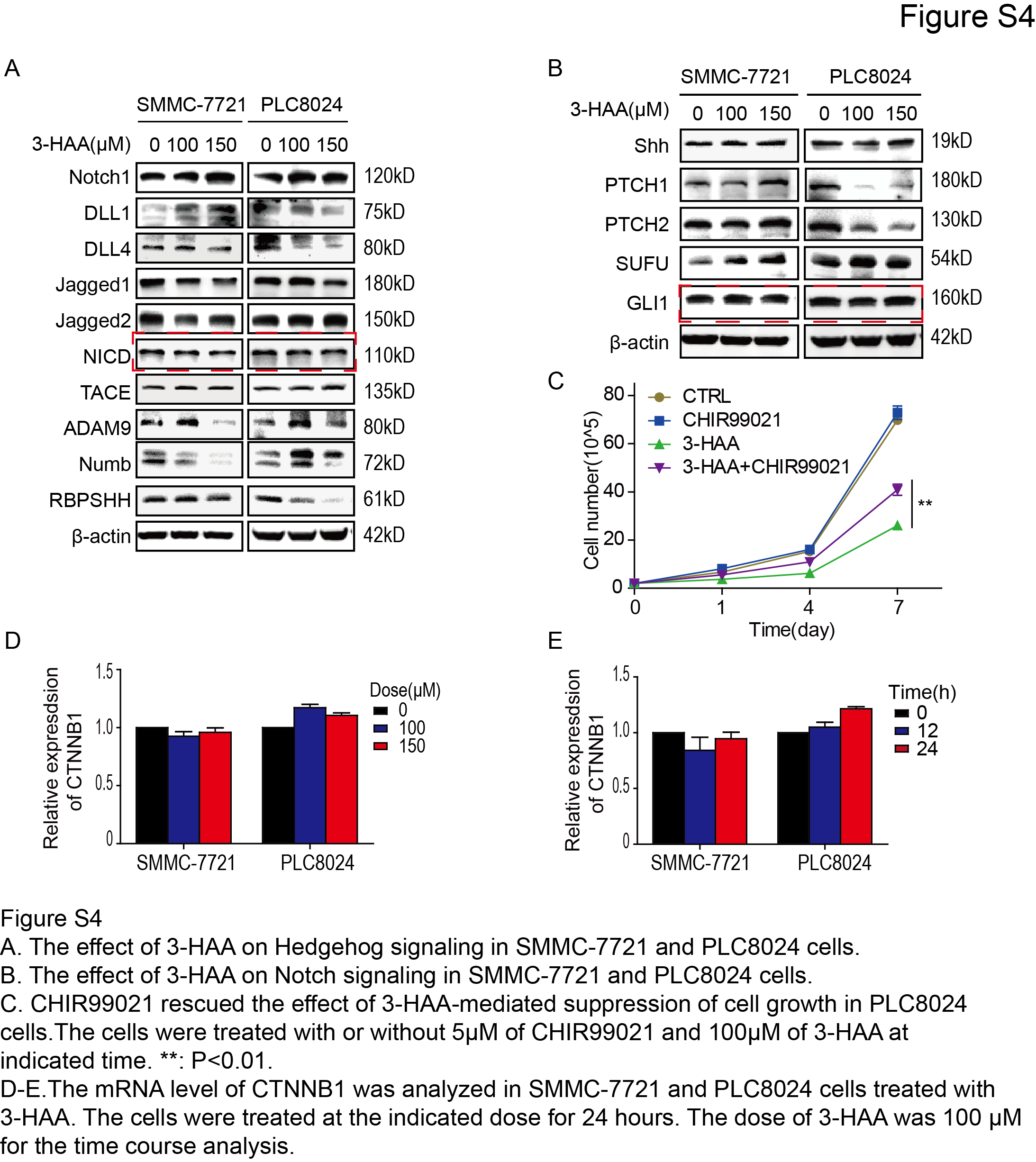

Supplement: Supplementary file 4 — Supplmental figure 4 [file 41420_2021_561_MOESM4_ESM.png]

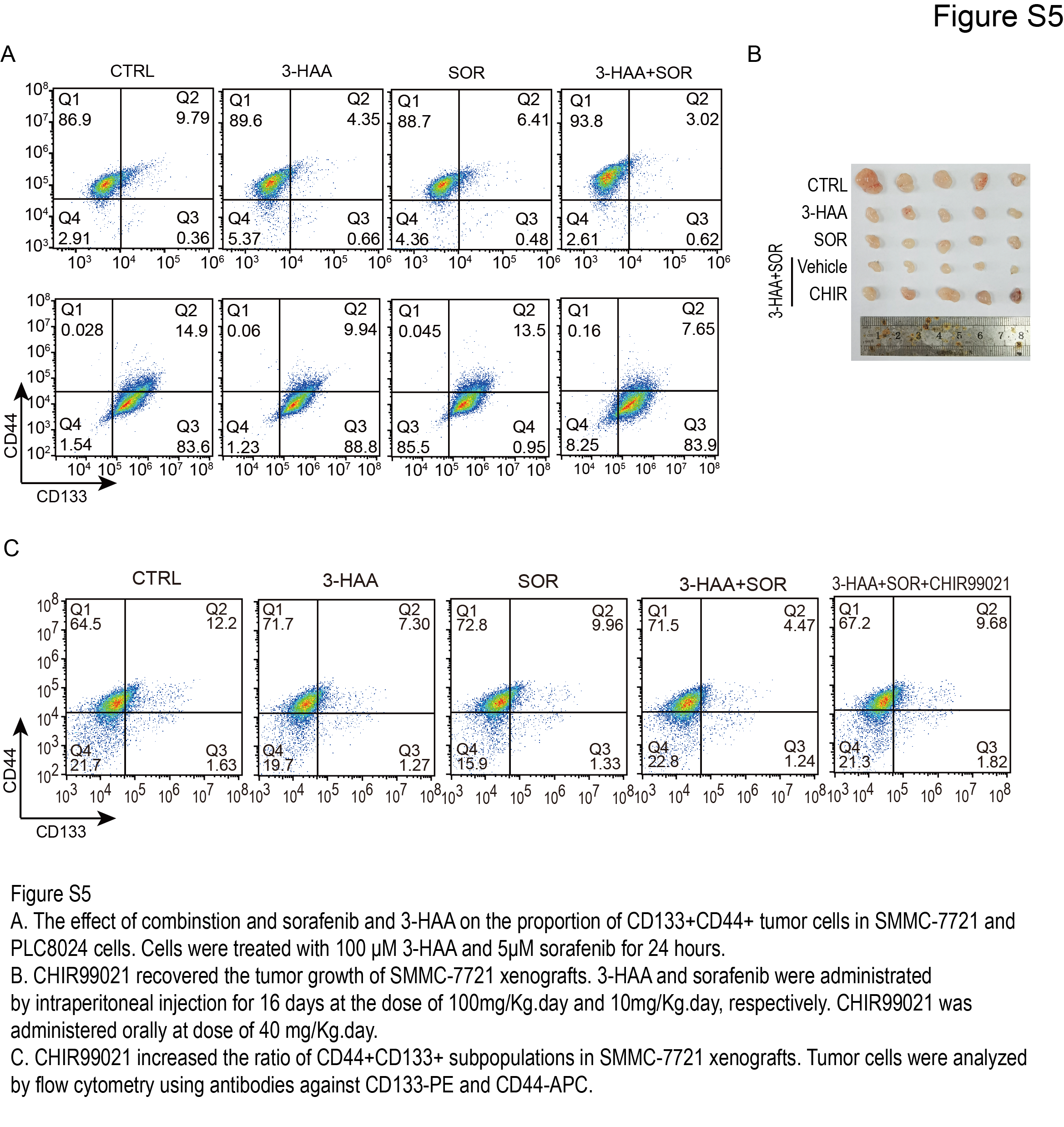

Supplement: Supplementary file 5 — Supplmental figure 5 [file 41420_2021_561_MOESM5_ESM.png]
